# Deep neural networks have an inbuilt Occam’s razor

**DOI:** 10.1038/s41467-024-54813-x

**Published:** 2025-01-14

**Authors:** Chris Mingard, Henry Rees, Guillermo Valle-Pérez, Ard A. Louis

**Affiliations:** 1 Rudolf Peierls Centre for Theoretical Physics, University of Oxford, Oxford, UK; 2 Physical and Theoretical Chemistry Laboratory, University of Oxford, Oxford, UK

**Keywords:** Computational science, Computer science

## Abstract

The remarkable performance of overparameterized deep neural networks (DNNs) must arise from an interplay between network architecture, training algorithms, and structure in the data. To disentangle these three components for supervised learning, we apply a Bayesian picture based on the functions expressed by a DNN. The prior over functions is determined by the network architecture, which we vary by exploiting a transition between ordered and chaotic regimes. For Boolean function classification, we approximate the likelihood using the error spectrum of functions on data. Combining this with the prior yields an accurate prediction for the posterior, measured for DNNs trained with stochastic gradient descent. This analysis shows that structured data, together with a specific Occam’s razor-like inductive bias towards (Kolmogorov) simple functions that exactly counteracts the exponential growth of the number of functions with complexity, is a key to the success of DNNs.

## Abstract (web summary)

Deep neural networks predict well despite having many more parameters than data points. The authors show that deep neural networks prefer simpler solutions due to an inbuilt “Occam’s razor”. Favouring simplicity avoids overfitting and captures patterns in data, explaining their success.

## Introduction

Although deep neural networks (DNNs) have revolutionized modern machine learning^1,2^, a fundamental theoretical understanding of why they perform so well remains elusive^3,4^. One of their most surprising features is that they work best in the overparameterized regime, with many more parameters than data points. As expressed in the famous quip: “*With four parameters I can fit an elephant, and with five I can make him wiggle his trunk.”* (attributed by Enrico Fermi to John von Neumann^5^), it is widely believed that having too many parameters will lead to overfitting: a model will capture noise or other inconsequential aspects of the data, and therefore predict poorly.

In statistical learning theory^6^ this intuition is formalized in terms of model capacity. It is not simply the number of parameters, but rather the complexity of the set of hypotheses a model can express that matters. The search for optimal performance is often expressed in terms of bias-variance trade-off. Models that are too simple introduce errors due to bias; they can’t capture the underlying processes that generate the data. Models that are too complex are over-responsive to random fluctuations in the data, leading to variance in their predictions.

DNNs are famously highly expressive^7–9^, i.e., they have extremely high capacity. Their ability to generalize therefore appears to break basic rules of statistical learning theory. Exactly how, without explicit regularization, DNNs achieve this feat is a fundamental question that has remained open for decades^3,4^. Although there has been much recent progress (see Supplementary Note 1 for a literature overview) there is no consensus for why DNNs work so well in the overparameterized regime.

Here we study this conundrum in the context of supervised learning for classification, where inputs *x*_*i*_ are attached to labels *y*_*i*_. Given a training set $S={\{\left.\right({x}_{i},{y}_{i}\}}_{i=1}^{m}$ of *m* input-output pairs, sampled i.i.d. from a data distribution ${{{\mathcal{D}}}}$, the task is to train a model on *S* such that it performs well (has low generalization error) when predicting output labels ${\hat{y}}_{i}$ for a test set *T* of unseen inputs, sampled from ${{{\mathcal{D}}}}$. For a DNN ${{{\mathcal{N}}}}({{\Theta }})$, with parameters ${{\Theta }}\subseteq {{\mathbb{R}}}^{p}$ (typically weights and biases), the accuracy on a training set can be captured by a loss function $L({\hat{y}}_{i},{y}_{i})$ that measures how close, for input *x*_*i*_, the prediction ${\hat{y}}_{i}$ of the DNN is to the true label *y*_*i*_. Training is typically done via some variant of stochastic gradient descent (SGD) which uses derivatives of $L({\hat{y}}_{i},{y}_{i})$ to adjust the parameters Θ in order to minimize the loss on *S*. Because DNNs are so highly expressive, and because SGD is typically a highly effective optimizer for DNNs, (near) zero training error (all correct labels after thresholding) on *S* is routinely achieved^7^.

## Functions and inductive bias

For classification, the question of why overparameterized DNNs don’t overfit can conveniently be expressed in terms of functions. For a given training set *S* and test set *T*, a function *f* can be defined on a restricted domain *S* + *T*. The inputs of *f* are the *x*_*i*_ ∈ *S* ∪ *T*, and the outputs include all possible sets of labels $\{{\hat{y}}_{i}\}$. Only one function gives the true labels {*y*_*i*_}. For a given set of parameters Θ, the DNN then represents a particular function *f*, which can be identified by the labels it outputs on the inputs *x*_*i*_ ∈ *S* ∪ *T*, after thresholding. Under the assumption that zero training error can be achieved, functions need only be distinguished by how they behave on the test set *T*. For *C* classes there are *N*_*T*_ = *C*^∣*T*∣^ possible functions *f* with zero error on the training set, this number is typically unimaginably large. The overwhelming majority of these functions will not generalize well. Since DNNs are highly expressive, they should be able to represent all (or nearly all) of these functions. The fundamental question of overparameterized DNN performance becomes a question of *inductive bias*: Why, from the unimaginably large set of functions that give zero error on *S*, do DNNs converge on a minuscule subset of functions that generalize well? Here, we will argue that a combination of structured data and specific Occam’s razor-like inductive bias towards simple functions which cancels out the exponential growth of the number of functions with increasing complexity helps answer this question.

## Distinguishing two questions about generalization

The main question we address here is what we call the *1st-order question of generalization* – Why do high-capacity learning models (such as DNNs) generalize at all? – This question of how inductive bias allows DNNs to break the conventional bias-variance trade-off expectations of classical learning theory has a long history. For example, it was famously highlighted in Leo Breiman’s 1995 commentary on refereeing for the NeurIPS conference^3^ (see also Appendix A) who phrased it by asking “Why don’t heavily overparameterised neural networks overfit the data?". While originally formulated for DNNs, the fact that infinite width limits of DNNs can reduce to neural network Gaussian processes (NNGPs)^10–12^, or kernels such as the Neural Tangent Kernel^13^, has stimulated a large volume of important theoretical work on GPs and kernels, see e.g.,^14,15^. In particular, these models recapitulate many properties of finite-width DNNs, including reaching small generalization errors on standard datasets such as CIFAR10^16,17^. While these methods are non-parametric, they have high capacity, and like DNNs^7^, can memorize random data^14^. For small capacity (or fewer parameters than data points for DNNs) all these models exhibit classic bias-variance trade-offs, with optimal generalization performance at intermediate capacity. However, as capacity (or the number of parameters) increases further, the generalization error markedly diminishes. This phenomenon, known as double-descent^18^, is observed in DNNs, kernels, and GPs, illustrating how these high-capacity models deviate from the conventional wisdom of classical statistical learning theory

The relative simplicity of GPs and kernels compared to DNNs has enabled the derivation of analytic estimates of the generalization error in terms of the kernel eigenfunctions and eigenvalues ^15,19–23^. Good generalization occurs when the kernel eigenfunctions with large eigenvalues align well with the target function being learned. Thus, these analyses offer a quantitative measure of precisely *how* the inductive bias of a high-capacity kernel must align with that of the learning task. However, they do not provide a broader explanation of the nature and origin of the inductive bias in these models, nor why it often matches the data they are trained on. That is the big question we will attempt to address here.

We want to disambiguate the broader 1st-order question above from a more specific 2nd-order question of generalization – Given a high capacity DNN that generalizes reasonably well (e.g., it solves the 1st order overparameterization/large capacity problem), can we understand how to improve its performance further?—This second question is crucial for deep learning practitioners: variations in architecture, hyperparameter tuning, data augmentation, etc., can significantly enhance performance over basic vanilla DNNs. However, these adjustments and tricks start from a base of a high-capacity model that already confounds expectations from classical learning theory. Because the two questions are sometimes conflated, we want to emphasize up front that this paper will focus on the 1st-order question, which is relevant to all high-capacity models. A better understanding of this fundamental question should help frame important second-order questions about further improving DNN performance.

## Learning Boolean functions: a model system

Inspired by calls to study model systems^3,4^, we first examine how a fully connected network (FCN) learns Boolean functions *f*: {0, 1}^*n*^ → {0, 1}, which are key objects of study in computer science. Just as the Ising model does for magnetism, this simple but versatile model allows us to capture the essence of the overparameterization problem, while remaining highly tractable. For a system of size *n*, there are 2^*n*^ inputs, and ${2}^{{2}^{n}}$ Boolean functions. Given a Boolean target function *f*_*t*_, the DNN is trained on a subset *S* of *m* < 2^*n*^ inputs, and then provides a prediction on a test set *T* which consists of the rest of the inputs. A key advantage of this system is that data complexity can easily be varied by choice of target function *f*_*t*_. Moreover, the model’s tractability allows us to calculate the prior *P*(*f*), likelihood, *P*(*S*∣*f*) and posterior *P*(*f*∣*S*) for different functions and targets, and so cast the tripartite schema of architecture, training algorithm, and structured data from^4^ into a textbook Bayesian picture.

## Results

### Quantifying inductive bias with Bayesian priors

The prior over functions, *P*(*f*), is the probability that a DNN ${{{\mathcal{N}}}}({{\Theta }})$ expresses *f* upon random sampling of parameters over a parameter initialization distribution *P*_par_(Θ):

1 $$P(f)=\int {\mathbb{1}}[{{{\mathcal{N}}}}({{\Theta }})==f]{P}_{{{{\rm{par}}}}}({{\Theta }})d{{\Theta }},$$

where ${\mathbb{1}}$ is an indicator function (1 if its argument is true, and 0 otherwise). Explicitly, this term is 1 if the neural network ${{{\mathcal{N}}}}({{\Theta }})$ expresses *f* with parameters Θ, else 0. It was shown in ref. ^24^ that, for ReLU activation functions, *P*(*f*) for the Boolean system was insensitive to different choices of *P*_par_(Θ), and that it exhibits an exponential bias of the form $P(f)\lesssim {2}^{-a\tilde{K}(f)+b}$ towards simple functions with low descriptional complexity $\tilde{K}(f)$, which is a proxy for the true (but uncomputable) Kolmogorov complexity. We will, as in ref. ^24^, calculate $\tilde{K}(f)$ using *C*_*LZ*_, a Lempel-Ziv (LZ) based complexity measure from ref. ^25^ on the 2^*n*^ long bitstring that describes the function, taken on an ordered list of inputs. Other complexity measures give similar results^24,26^, so there is nothing fundamental about this particular choice. To simplify notation, we will use *K*(*f*) instead of $\tilde{K}(f)$. The exponential drop of *P*(*f*) with *K*(*f*) in the map from parameters to functions is consistent with an algorithmic information theory (AIT) coding theorem^27^ inspired *simplicity bias* bound^25^ which works for a much wider set of input-output maps. It was argued in ref. ^24^ that if this inductive bias in the priors matches the simplicity of structured data then it would help explain why DNNs generalize so well. However, the weakness of that work, and related works arguing for such a bias towards simplicity^24,26,28–35^, is that it is typically not possible to significantly change this inductive bias towards simplicity, making it hard to conclusively show that it is not some other property of the network that instead generates the good performance. Here we exploit a particularity of $\tanh$ activation functions that enable us to significantly vary the inductive bias of DNNs. In particular, for a Gaussian *P*_par_(Θ) with standard deviation *σ*_*w*_, it was shown^36,37^ that, as *σ*_*w*_ increases, there is a transition to a chaotic regime. Moreover, it was recently demonstrated that the simplicity bias in *P*(*f*) becomes weaker in the chaotic regime^38^ (see also Supplementary Note 3). We will exploit this behavior to systematically vary the inductive bias over functions in the prior.

**Fig. 1:**
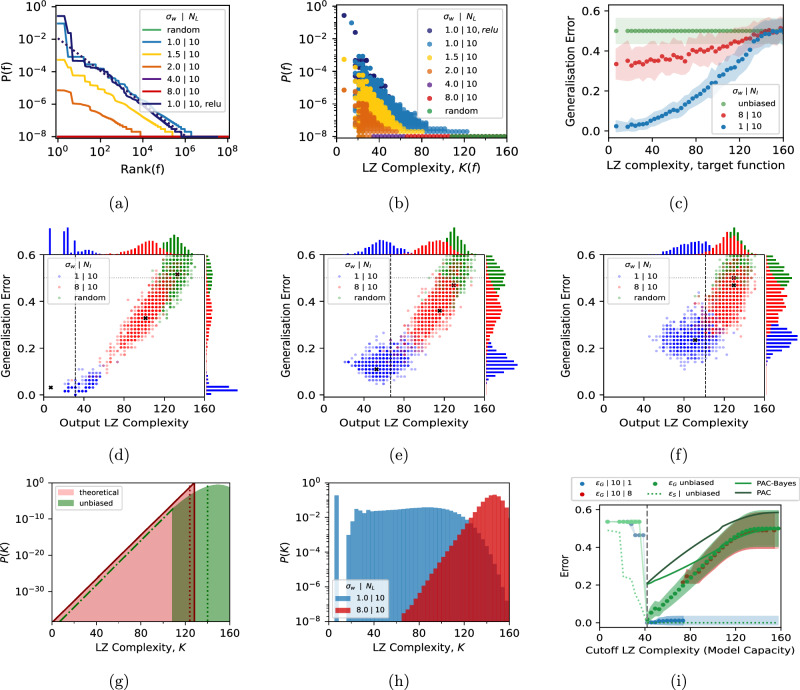
Priors over functions and over complexity. **a** Prior *P*(*f*) that a *N*_*l*_-layer FCN with $\tanh$ activations generates *n* = 7 Boolean functions *f*, ranked by probability of individual functions, generated from 10^8^ random samples of parameters Θ over a Gaussian *P*_par_(Θ) with standard deviations *σ*_*w*_ = 1…8. Also compared is a ReLU-activated DNN. The dotted blue line denotes a Zipf’s law prior^24^ $P(f)=1/((128\ln 2)Rank(f))$. **b** *P*(*f*) versus LZ complexity *K* for the networks from (**a**). **c** generalization error versus *K* of the target function for an unbiased learner (green), and ${\sigma }_{w}=1,8\,\tanh$ networks trained to zero error with advSGD^24^ on cross-entropy loss with training set *S* of size *m* = 64, for 1000 random initializations. The error is calculated on the remaining ∣*T*∣ = 64 functions. Error bars are one standard deviation (See Fig. S17 for PAC-Bayes bounds on this data). **d**, **e**, **f** Scatterplots of generalization error versus learned function LZ complexity, from 1000 random initializations for three target functions from subfigure (**c**). The dashed vertical line denotes the target function complexity. The black cross represents the mode function. The histograms at the top (side) of the plots show the posterior probability upon training as a function of complexity,*P*_SGD_(*K*∣*S*) (error,*P*_SGD_(*ϵ*_*G*_∣*S*)). **g** The prior probability *P*(*K*) to obtain a function of LZ complexity *K* for uniform random sampling of 10^8^, compared to a theoretical perfect compressor. 90% of the probability mass lies to the right of the vertical dotted lines, and the dash-dot line denotes an extrapolation to low *K*. **h** *P*(*K*) is relatively uniform on *K* for the *σ*_*w*_ = 1 system, while it is highly biased towards complex functions for the *σ*_*w*_ = 8 networks. The large difference in these priors helps explain the significant variation in DNN performance. **i** generalization error for the K-learning restriction for the *σ*_*w*_ = 1, 8 DNNs and for an unbiased learner, all for ∣*S*∣ = 100. *ϵ*_*S*_ is the training error and *ϵ*_*G*_ is the generalization error on the test set. The vertical dashed line is the complexity *K*_*t*_ of the target. Also compared are the standard realizable PAC and marginal-likelihood PAC-Bayes bounds for the unbiased learner. In 10^4^ samples, no solutions were found with *K* ≲ 70 for the *σ*_*w*_ = 8 DNN, and with *K* ≳ 70 for the *σ*_*w*_ = 1 DNN.

In Fig. 1a, b we depict prior probabilities *P*(*f*) for functions *f* defined on all 128 inputs of a *n* = 7 Boolean system upon random sampling of parameters of an FCN with 10 layers and hidden width 40 (which is provably fully expressive for this system^31^), and $\tanh$ activation functions. The simplicity bias in *P*(*f*) becomes weaker as the width *σ*_*w*_ of the Gaussian *P*_par_(*σ*_*w*_) increases. By contrast, for ReLU activations, the bias in *P*(*f*) barely changes with *σ*_*w*_ (see Fig. S3a). The effect of the decrease in simplicity bias on DNN generalization performance is demonstrated in Fig. 1c for a DNN trained to zero error on a training set *S* of size *m* = 64 using advSGD (an SGD variant taken from ref. ^24^), and tested on the other 64 inputs *x*_*i*_ ∈ *T*. The generalization error (the fraction of incorrect predictions on *T*) varies as a function of the complexity of the target function. Although all these DNNs exhibit simplicity bias, weaker forms of the bias correspond to significantly worse generalization on the simpler targets (see also Supplementary Note 10). For very complex targets, both networks perform poorly. For reference, we also show an unbiased learner, where functions *f* are chosen uniformly at random with the proviso that they exactly fit the training set *S*. Not surprisingly, given the 2^64^ ≈ 2 × 10^19^ functions that can fit *S*, the performance of this unbiased learner is no better than random chance.

The scatter plots of Fig. 1d–f depict a more fine-grained picture of the behavior of the SGD-trained networks for three different target functions. For each target, 1000 independent initializations of the SGD optimizer, with initial parameters taken from *P*_par_(*σ*_*w*_), are used. The generalization error and complexity of each function found when the DNN first reaches zero training error are plotted. Since there are 2^64^ possible functions that give zero error on the training set *S*, it is not surprising that the DNN converges to many different functions upon different random initializations. For the *σ*_*w*_ = 1 network (where *P*(*f*) resembles that of ReLU networks) the most common function is typically simpler than the target. By contrast, the less biased network converges on functions that are typically more complex than the target. As the target itself becomes more complex, the relative difference between the two generalization errors decreases, because the strong inductive bias towards simple functions of the first network becomes less useful. No free lunch theorems for supervised learning tell us that when averaged over all target functions, the three learners above will perform equally badly^39,40^ (see also Supplementary Note 43).

### Priors over complexity

To understand why relatively modest changes in the inductive bias towards simplicity lead to such significant differences in generalization performance, we need another important ingredient, namely how the *number* of functions vary with complexity. Basic counting arguments imply that the number of strings of a fixed length that have complexity *K* scales exponentially as 2^*K*^ ^27^. Therefore, the vast majority of functions picked at random will have high complexity. This exponential growth of the number of functions with complexity can be captured in a more coarse-grained prior, the probability *P*(*K*) that the DNN expresses a function of complexity *K* upon random sampling of parameters over a parameter initialization function *P*_par_(Θ), which can also be written in terms of functions as $P({K}^{{\prime} })={\sum }_{f\in {{{{\mathcal{H}}}}}_{{K}^{{\prime} }}}P(f)$, the weighted sum over the set ${{{{\mathcal{H}}}}}_{{K}^{{\prime} }}$ of all functions with complexity $\tilde{K}(f)={K}^{{\prime} }$. In Fig. 1g *P*(*K*) is shown for uniform random sampling of functions for 10^8^ samples using the LZ measure, and also for the theoretical ideal compressor with $P(K)={2}^{K-{K}_{max}-1}$ over all 2^128^ ≈ 3 × 10^38^ functions (see also Supplementary Note 9). In (h) we display *P*(*K*) for functions not sampled at random, but rather from the two networks. There is a dramatic difference between random sampling functions (as in (g)) and between the network with *σ*_*w*_ = 1, where *P*(*K*) is nearly flat. This behavior follows from the interesting fact that the AIT coding theorem-like scaling^24,25^ of the prior over functions $P(f) \sim {2}^{-\tilde{K}(f)}$ counters the 2^*K*^ growth in the number of functions.

By contrast, even though, relative to the 38 or so orders of magnitude scale on which *P*(*f*) varies, the more artefactual *σ*_*w*_ = 8 system has strong simplicity bias (we estimate that for the simplest functions, *P*(*f*) is about 10^25^ times higher than the mean probability $< P(f) > ={2}^{-128}\approx 3\times 1{0}^{-39}$), this is not enough to counter the 2^*K*^ growth in the number of functions with complexity. Therefore, this DNN is exponentially more likely to throw up complex functions, an effect that SGD is unable to overcome.

More generally, the fact that the number of complex functions grows exponentially with complexity *K* lies at the heart of the classical explanation of why an insufficiently biased agent suffers from variance: It can too easily find many different functions that all fit the data. The marked differences in the generalization performance between the two networks observed in Fig. 1c–f can be therefore traced to differences in the inductive bias of the networks, as measured by the differences in their priors.

### Artificially restricting model capacity

To further illustrate the effect of inductive bias we create a K-learner that only allows functions with complexity ≤*K*_*M*_ to be learned and discards all others. As can be seen in Fig. 1i, the learners typically cannot reach zero training error on the training set if *K*_*M*_ is less than the target function complexity *K*_*t*_. For *K*_*M* _≥ *K*_*t*_, zero training error can be reached and not surprisingly, the lowest generalization error occurs when *K*_*M*_ = *K*_*t*_. As the upper limit *K*_*M*_ is increased, all three learning agents are more likely to make errors in predictions due to variance. The random learner has an error that grows linearly with *K*_*M*_. This behavior can be understood with a classic Probably Approximately Correct (PAC) bound^6^ where the generalization error (with confidence 0 ≤ (1 − *δ*) ≤1) scales as ${\epsilon }_{G}\le (\ln | {{{{\mathcal{H}}}}}_{\le {K}_{M}}| -\ln \delta )/m$, where $| {{{{\mathcal{H}}}}}_{\le {K}_{M}}| \,K\le {K}_{M}$ is the size of the hypothesis class of all functions with *K*≤*K*_*M*_; the bound scales linearly in *K*_*M*_, as the error does (see Supplementary Note 7 for further discussion including the more sophisticated PAC-Bayes bound^41,42^). The generalization error for the *σ*_*w*_ = 1 DNN does not change much with *K*_*M*_ for *K*_*M*_ > *K*_*t*_ because the strong inductive bias towards simple solutions means access to higher complexity solutions doesn’t significantly change what the DNN converges on.

Finally, we show data for DNNs in the ordered regime with *σ*_*w*_ ≪ 1, and for other optimizers, loss functions, and activation functions in Figs. S6–S11. These results broadly exhibit the same behavior we describe here.

### Calculating the Bayesian posterior and likelihood

To better understand the generalization behavior observed in Fig. 1 we apply Bayes’ rule, *P*(*f*∣*S*) = *P*(*S*∣*f*)*P*(*f*)/*P*(*S*) to calculate the Bayesian posterior *P*(*f*∣*S*) from the prior *P*(*f*), the likelihood *P*(*S*∣*f*), and the marginal likelihood *P*(*S*). Since we condition on zero training error, the likelihood takes on a simple form. *P*(*S*∣*f*) = 1if ∀ *x*_*i*_ ∈ *S*, *f*(*x*_*i*_) = *y*_*i*_, while *P*(*S*∣*f*) = 0 otherwise. For a fixed training set, all the variation in *P*(*f*∣*S*) for *f* ∈ *U*(*S*), the set of all functions compatible with *S*, comes from the prior *P*(*f*) since *P*(*S*) is constant. Therefore, in this Bayesian picture, the bias in the prior is translated over to the posterior.

The marginal likelihood also takes a relatively simple form for discrete functions, since *P*(*S*) = ∑_*f*_*P*(*S*∣*f*)*P*(*f*) = ∑_*f*∈*U*(*S*)_*P*(*f*). It is equivalent to the probability that the DNN obtains zero error on the training set *S* upon random sampling of parameters, and so can be interpreted as a measure of the inductive bias towards the data. The Marginal-likelihood PAC-Bayes bound^42^ makes a direct link $P(S)\lesssim {e}^{-m{\epsilon }_{G}}$ to the generalization error *ϵ*_*G*_ which captures the intuition that, for a given *m*, a better inductive bias towards the data (larger *P*(*S*)) implies better performance (lower *ϵ*_*G*_).

One can also define the posterior probability *P*_SGD_(*f*∣*S*), that a network trained with SGD (or another optimizer) on training set *S*, when initialized with *P*_par_(Θ), converges on function *f*. For simplicity, we take this probability at the epoch where the system first reaches zero training error. Note that in Fig. 1d–f it is this SGD-based posterior that we plot in the histograms at the top and sides of the plots, with functions grouped either by complexity, which we will call *P*_SGD_(*K*∣*S*), or by generalization error *ϵ*_*G*_, which we will call *P*_SGD_(*ϵ*_*G*_∣*S*).

DNNs are typically trained by some form of SGD, and not by randomly sampling over parameters which is much less efficient. However, a recent study^43^ which carefully compared the two posteriors has shown that to first order, *P*_B_(*f*∣*S*) ≈ *P*_SGD_(*f*∣*S*), for many different data sets and DNN architectures. We demonstrate this close similarity in Fig. S15 explicitly for our *n* = 7 Boolean system. This evidence suggests that Bayesian posteriors calculated by random sampling of parameters, which are much simpler to analyze, can be used to understand the dominant behavior of an SGD-trained DNN, even if, for example, hyperparameter tuning can lead to 2nd-order deviations between the two methods (see also Supplementary Note 1).

To test the predictive power of our Bayesian picture, we first define the function error *ϵ*(*f*) as the fraction of incorrect labels *f* produces on the full set of inputs. Next, we average Bayes’ rule over all training sets *S* of size *m*:

2 $${\langle P(f| S)\rangle }_{m}=P(f){\left\langle \frac{P(S| f)}{P(S)}\right\rangle }_{m}\approx \frac{P(f){\left(1-\epsilon (f)\right)}^{m}}{{\langle P(S)\rangle }_{m}}$$

where the mean likelihood 〈*P*(*S*∣*f*)〉_*m*_ = (1−*ϵ*(*f*))^*m*^ is the probability of a function *f* obtaining zero error on a training set of size *m*. In the second step, we approximate the average of the ratio with the ratio of the averages which should be accurate if *P*(*S*) is highly concentrated, as is expected if the training set is not too small.

Equation (2) is hard to calculate, so we coarse-grain it by grouping together functions by their complexity:

3 $${\langle P(K| S)\rangle }_{m}={\sum}_{{C}_{LZ}(f)=K}{\langle P(f| S)\rangle }_{m}\propto P(K){\langle {\left(1-{\epsilon }_{G}(K)\right)}^{m}\rangle }_{l},$$

and in the second step make a *decoupling approximation* where we average the likelihood term over a small number*l*of functions with complexity *K* with lowest generalization error *ϵ*_*G*_(*K*) since the smallest errors in the sum dominate exponentially since $(1-{\epsilon }_{G})\approx {e}^{-{\epsilon }_{G}}$ for ∣*ϵ*_*G*_∣ ≪ 1. We then multiply by *P*(*K*), which takes into account the value of the prior and the multiplicity of functions at that *K*, and normalize ∑_*K*_*P*(*K*∣*S*) = 1. For a given target, we make the ansatz that this decoupling approximation provides an estimate that scales as the true (averaged) posterior.

**Fig. 2:**
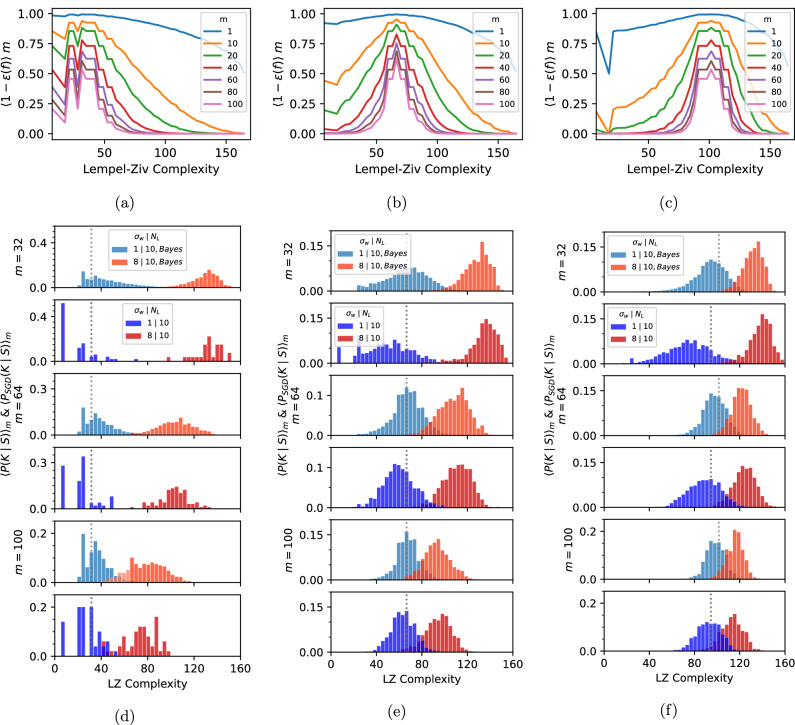
How training data affects the posteriors. **a**–**c** depict the mean likelihood ${\langle {(1-{\epsilon }_{G}(K))}^{m}\rangle }_{5}$ from Equation (3), averaged over training sets, and over the 5 lowest error functions at each *K*. This term depends on data and is independent of the DNN architecture. With increasing *m* it peaks more sharply around the complexity of the target. In (**d**–**f**) we compare the posteriors over complexity, ${\langle {P}_{{{{\rm{SGD}}}}}(K| S)\rangle }_{m}$, for SGD (darker blue and red) averaged over training sets of size *m*, to the prediction of 〈*P*(*K*∣*S*)〉_*m*_ from Equation (3) (lighter blue and orange), calculated by multiplying the Bayesian likelihood curves in (**a**–**c**) by the prior *P*(*K*) shown in Fig. 1h. The light (Bayes) and dark (DNN) blue histograms are from the *σ*_*w*_ = 1 system, and the orange (Bayes) and red (DNN) histograms are from the *σ*_*w*_ = 8 system which has less bias towards simple functions. The Bayesian decoupling approximation (Equation (3)) captures the dominant trends in the behavior of the SGD-trained networks as a function of data complexity and training set size. Quantitative measures of the similarity between the posteriors can be found in Fig. S22.

To test our approximations, we first plot, in Fig. 2a–c, the likelihood term in Equation (3) for three different target functions. To obtain these curves, we considered a large number of functions (including all functions with up to 5 errors w.r.t. the target, with further functions sampled). For each complexity, we average this term over the *l* = 5 functions with smallest *ϵ*_*G*_. Not surprisingly, functions close to the complexity of the target have the smallest error. These graphs help illustrate how the DNN interacts with data. As the training set size *m* increases, functions that are most likely to be found upon training to zero training error are increasingly concentrated close to the complexity of the target function.

To test the decoupling approximation from Eq. (3), we compare in Fig. 2d–f the posterior 〈*P*(*K*∣*S*)〉_*m*_, calculated by multiplying the Bayesian likelihood curve from Fig. 2a–c with the two Bayesian priors *P*(*K*) from Fig. 1h, i, to the posteriors ${\langle {P}_{rmSGD}(K| S)\rangle }_{m}$ calculated by advSGD^24^ over a 1000 different parameter initializations and training sets. It is remarkable to see how well the simple decoupling approximation performs across target functions and training set sizes. In Figs. S13 and S14 we demonstrate the robustness of our approach by showing that using *l* = 1 or *l* = 50 functions does not change the predictions much. This success suggests that our simple approach captures the essence of the interaction between the data (measured by the likelihood, which is independent of the learning algorithm), and the DNN architecture (which is measured by the prior and is independent of the data).

We have therefore separated out two of the three parts of the tripartite scheme, which leaves the training algorithm. In the figures above our Bayesian approximation captures the dominant behavior of an SGD-trained network. This correspondence is consistent with the results and arguments of ref. ^43^. We checked this further in Fig. S15 for a similar set-up using MSE loss, where Bayesian posteriors can be exactly calculated using Gaussian processes (GPs). The direct Bayesian GP calculation closely matches SGD-based results for our much smaller network. Note that, in the spirit of model calculations, as called for in ref. ^3,4^, we mainly used a much smaller DNN. But their agreement with the GP-based posteriors, calculated for the infinite width limit, shows that at the scale of our Bayesian approach to the 1st-order generalization question we are addressing here, the size of the DNN is not an important factor. The width of a DNN can, of course, be a factor for 2nd order generalization questions.

### Beyond the Boolean model: MNIST & CIFAR-10

**Fig. 3:**
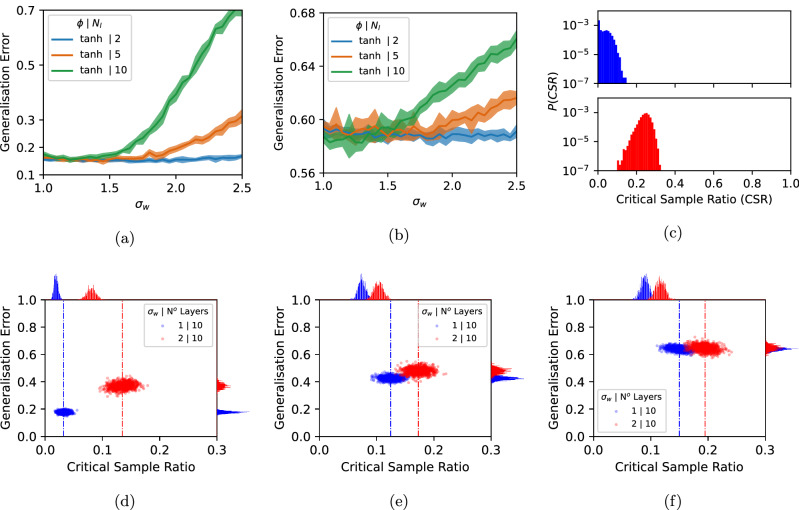
MNIST and CIFAR-10 data. **a** MNIST generalization error for FCNs on a 1000 image training set versus *σ*_*w*_ for three depths. **b** CIFAR10 generalization error for FCNs trained on a 5000 image training set versus *σ*_*w*_ for three depths. The FCNs, made of multiple hidden layers of width 200, were trained with SGD with batch size 32 and lr=10^−3^ until 100% accuracy was first achieved on the training set. Error bars are one standard deviation. **c** Complexity prior *P*(*K*), for CSR complexity, for 1000 MNIST images for randomly initialized networks of 10 layers and *σ*_*w*_ = 1, 2. Probabilities are estimated from a sample of 2 × 10^4^ parameters. **d**, **e**, **f** are scatterplots of generalization error versus the CSR for 1000 networks trained to 100% accuracy on a training set of 1000 MNIST images and tested on 1000 different images. In (**d**) the training labels are uncorrupted, in (**e**, **f**) 25% and 50% of the training labels are corrupted respectively. Note the qualitative similarity to the scatter plots in Fig. 1d–f.

Can the principles worked out for the Boolean system be observed in larger systems that are closer to the standard practice of DNNs? To this end, we show, in Fig. 3a, b how the generalization error for the popular image datasets MNIST and CIFAR-10 changes as a function of the initial parameter width *σ*_*w*_ and the number of layers *N*_*l*_ for a standard FCN, trained with SGD on cross-entropy loss with $\tanh$ activation functions. Larger *σ*_*w*_ and larger *N*_*l*_ push the system deeper into the chaotic regime^36,37^ and result in decreasing generalization performance, similar to what we observe for the Boolean system for relatively simple targets. In Fig. 3, we plot the prior over complexity *P*(*K*) for a complexity measure called the critical sample ratio (CSR)^28^, an estimate of the density of decision boundaries that should be appropriate for this problem. Again, increasing *σ*_*w*_ greatly increases the prior probability that the DNN produces more complex functions upon random sampling of parameters. Thus the decrease in generalization performance is consistent with the inductive bias of the network becoming less simplicity biased, and therefore less well aligned with structure in the data. Indeed, datasets such as MNIST and CIFAR-10 are thought to be relatively simple^44,45^.

These patterns are further illustrated in Fig. 3d–f where we show scatterplots of generalization error v.s. CSR complexity for three target functions that vary in complexity (here obtained by corrupting labels). The qualitative behavior is similar to that observed for the Boolean system in Fig. 1. The more simplicity-biased networks perform significantly better on the simpler targets, but the difference with the less simplicity-biased network decreases for more complex targets. While we are unable to directly calculate the likelihoods because these systems are too big, we argue that the strong similarities to our simpler model system suggest that the same basic principles of inductive bias are at work here.

## Discussion

In order to generalize, high capacity models need a clear inductive bias towards functions that perform well on the data being studied^46^. Here we show that DNNs and also NNGPs and DNN-inspired kernels have a very specific kind of inbuilt Occam’s razor (see Supplementary Note 4 for background on Occam’s razor(s)). This inductive bias can be quantified by an AIT coding theorem-like scaling of the prior as *P*(*f*) ∝ 2^−*K*(*f*)^ which can counteract the 2^*K*^ growth of the number of functions with complexity. If this intrinsic inductive bias is slightly weaker, say *P*(*f*) ∝ 2^−*αK*(*f*)^ with *α* < 1, then it becomes much harder to overcome the 2^*K*^ growth and the learner will likely suffer from strong variance problems. While we were not able to significantly increase the bias towards simplicity in DNNs, too strong a bias towards simplicity can mean bias (instead of variance) problems because complex functions become hard to find^26,47^.

An interesting direction to explore is the more formal arguments in AIT relating to the optimality of Solomonoff induction^27,48,49^ (see also Supplementary Note 44). In particular, there may be fruitful links between simplicity bias, Solomonoff induction, and compression in deep learning, as recently discussed for example in the context of large language models^50,51^.

Another promising direction for exploration is to connect our high-level theory here with the more detailed calculations of generalization in kernels^19–23,15^. These works can tell us *how* DNN inspired kernels need to align with data, but not so easily *why* this would be so. An extra challenge is that our work here has been in the context of discrete functions and classification, whereas the work on kernels is typically for continuous regression scenarios. However, the classification setting allows us to study the inductive bias of a model over the entire space of input functions and simultaneously make concrete generalization bounds. We distinguished the first-order question of why high-capacity DNNs (and related models) generalize at all from the second-order question of how to further improve DNN performance. Firstly, the fact that DNNs are strongly biased towards simplicity gives a basis upon which to look for further inductive biases in other directions that may be orthogonal to simplicity. Our results are derived in a Bayesian setting, but do not preclude the fact that SGD optimization itself can also introduce useful inductive biases (see also Supplementary Note 1). In particular, SGD likely enables feature learning. Our understanding of how this works, even in the infinite width kernel or GP limit^52–54^, remains in early stages, but feature-learning is likely to play an important role in explaining why DNNs outperform other methods on large datasets.

Much of the exciting recent progress in foundation models such as GPT-4 likely arises from optimizer-induced inductive biases beyond the simplicity bias at initialization that may also play an important role there^26^. Direct inspiration from biology can also provide inspiration for alternate inductive biases, as recently shown for image recognition in CNNs^55^. Systematically studying and understanding how these inductive biases interact with data remains one of the key challenges in modern machine learning.

Finally, our observations about inductive bias can be inverted to identify characteristics—such as limits on the complexity – of the data that DNNs can successfully learn. In particular, the remarkable success of DNNs on a broad range of scientific problems^56–59^ suggests that their inductive biases must recapitulate something deep about the structure of the natural world^29^. By understanding why DNNs select specific solutions, we may, in turn, gain profound insights into the structure of nature itself ^60^.

## Supplementary information

Supplementary Information

Transparent Peer Review file

## Data Availability

All code to generate the data used in this study is publicly available under the CC-BY 4.0 license  in the following zenodo repository 10.5281/zenodo.13997032.

## Code availability

All code to generate the figures in this study is publicly available in the following zenodo repository 10.5281/zenodo.13997032.
