# Supplementary material for: Deep neural networks have an inbuilt Occam’s razor
Source: Nat Commun. 2025 Jan 14;16:220. doi: 10.1038/s41467-024-54813-x (PMC11733143; doi:10.1038/s41467-024-54813-x)
Supplement: Supplementary file 2 — Transparent Peer Review file [file 41467_2024_54813_MOESM2_ESM.pdf]

nature portfolio

Peer Review File

Deep neural networks have an inbuilt Occam's razor

Corresponding Author: Professor Ard Louis

This file contains all reviewer reports in order by version, followed by all author rebuttals in order by version.

Version 0:

Reviewer comments:

Reviewer #2

(Remarks to the Author)
This paper tries to argue that the deep neural networks have an inbuilt Occam's razor, from the perspective of Bayesian picture. Specifically, the authors considers the Boolean function setting, where the target function f maps Boolean inputs {0,1}^n to {0,1}, and consider the case with Bayesian priors P(f). They want to understand that if the neural networks are defined on 7 Boolean variables, are 10-layer feedforward network with hidden width 40, and the parameters are randomly sampled, what will happen. Then in Figure 1 and Figure 2, they run experiments to demonstrate some relationships between priors and complexities of the function, as well as how training data affects the posteriors.

Honestly speaking, I think this paper is very far away from the bar of Nature communication. First, I am pretty sure that the functions that neural networks learn are not Boolean functions. Moreover, Boolean functions should take real values for outputs, i.e., [0,1], not {0,1} as assumed in the paper. So the settings the authors considered are extremely restricted. Secondly, the 7 Boolean-variables, 10-layer, 40 hidden width setting is extremely unrealistic, and nobody uses such structure empirically. I think even 8 years ago, such assumptions will be considered extremely strong for submissions to ICML/NeurIPS. Moreover, this is a purely experimental paper, without any solid evidence of showing why the claims of the authors are true. In fact, it is a bit difficult for me to find the exact claims of the authors. Based on the introduction, I think the authors want to demonstrate that the neural network has an inherent selection over simple models during the training process. This is a very important problem, but I do not think this paper provides any convincing answers to it.

In the last two pages, the authors also provide some preliminary results on MNIST/CIFAR-10, for experimental results with FCNs. I do not think these results are solid enough to support their claims.

To make it clear, I think the main problem of this paper, is the authors picked the wrong title. The correct title should be, “empirically, do 10-layer 40 hidden width feedforward networks on 7 Boolean variables have evidence showing inbuilt Occam's razor?” I think this is what the paper is really about, which is not significant enough.

Reviewer #3

(Remarks to the Author)
This manuscript makes a mathematical argument, couched in a Bayesian framework, for Deep Neural Networks (DNNs) having an inductive bias such that the functions they are predisposed to learn shrink in probability exponentially with increasing complexity, which counteracts the exponential growth in the space of possible functions as their complexity grows. This argument is instantiated with simulations demonstrating the effect, measured in terms of the probability of learning a particular function and the generalisation error, on some toy model architectures applied to simple boolean and standard benchmark datasets (i.e. CIFAR-10 and MNIST), as some of their key parameters, such as depth, are systematically varied.

This is a noteworthy and insightful result shedding light on a complex and powerful property of DNNs, relevant to the perplexing findings identified in such work as Nakkiran et al., 2019 (https://arxiv.org/abs/1912.02292); a publication which this manuscript would benefit from citing. The approach appears to be methodologically sound, however many of the justifications for the simplifying assumptions and central tenets of the work are relegated to the appendix - if the journal's restrictions permit, the manuscript would benefit by further substantiating some of these assumptions in the main text.

Regarding the separate question of how to improve the generalisation of DNNs, the authors state: "Understanding this basic problem should help frame important 2nd-order questions about how to improve DNN performance further." While they take care to disentangle these questions in the introduction, having arrived at an understanding of the basic problem, the authors do not discuss this aspect, which would be useful for the large proportion of readers interested in the "second-order question" - how has their finding helped to frame such questions and what insights into how to further improve DNN performance has it or could it provide?

There are also a few ways in which the rigour of the analysis could be improved, such as in Figure 2(d)-(f) on p5, it would strengthen the argument that the Bayes and DNN histograms are similar if a suitable statistical test showed no significant difference.

There are also a few small mistakes and typos which should be corrected, as follows.

* "its argument is true if the parameters of N (Θ) are such that represents f It was shown in [10] that", p2: this is ungrammatical and missing a full stop.
* Please make the ordering of items in the legends consistent e.g. 1(a) and (b), p3.
* "PAC bound" p4: Define acronyms on first use.
* "(with confidence $0 \geq (1 - \delta) \leq 1$)" p4: the first inequality appears to be reversed.
* "(Eq. (3) captures the dominant trends...": Missing bracket in Fig 2, p5.
* "Beyond the Boolean model: MINST & CIFAR-10", p6: Typo in MNIST.
* "2nd order question of fine-tuned generalization": There is a formatting anomaly half way down p2 of the Supplementary materials.

Overall, I find this to be an important analysis of DNNs and subject to the revisions above, I recommend it for publication.

Version 2:

Reviewer comments:

Reviewer #2

(Remarks to the Author)
I have read the authors' rebuttal. However, I did not change my mind.

1. Sorry, I still do not think neural network can be approximated with Boolean functions. I have read many theory papers on deep learning, and I have never seen such assumptions previously. I think this is a very strong assumption that I cannot accept.

2. Sorry, Boolean functions take real values. Please check https://arxiv.org/abs/2105.10386 by Ryan O'Donnell. It is standard to consider Boolean function outputs as real value, not discrete value.

3. Sorry, I think the 7 Boolean-variables, 10-layer, 40 hidden width setting is extremely unrealistic, and nobody uses such structure empirically. I am afraid that I cannot agree with the authors that this case captures enough details of the topic.

I also read the rest of the rebuttal, and did not change my mind.

Therefore, I will maintain my score unchanged. I think this paper is very far away from being published at Nature communications.

Reviewer #3

(Remarks to the Author)
As per my previous review, I consider that this article addresses an interesting and important point in a rigorous and controlled way. Unlike the other reviewer, I like the authors' approach of paring the problem down to a toy problem (at least, relative to the cutting edge of the datasets DNNs are typically trained on) but one that is tractable, finely controllable and in their own words "foundational". Indeed, it is hard to argue that boolean logic is anything but foundational in computer science.

However, to help bridge the gap between the experimental petri disk of boolean functions and the more complex problems of engineering applications, (in order to counter the objections such as those faced in this review process from Rev. 2.) it would help to give more prominence to the studies (in the main text) where the authors scaled the problem up and found similar results for the basic behaviours.

To further assuage such objections, the introduction could be slightly expanded with citations to more applied work on inductive biases in DNN. For example, in a biologically-inspired convolutional neural network, less was also shown to be more (i.e. through simplifications/constraints conferring benefits) in work by Evans et al. (2022) whereby fixing the form of the first layer convolutional kernels led to greater robustness to noise and generalisation on out-of-distribution images. Inductive biases of this sort found through evolution may similarly have been implicitly found by engineers tinkering with neural networks (prior to this analytical perspective), contributing to the enormous popularity and success they have enjoyed.

A brief summary of the central hypothesis of the authors' forthcoming paper on feature learning could also be included in this section. I appreciate that the full story can not be included in the current paper but a sentence or two to at least articulate the central question and sketch out their idea (citing the manuscript in preparation for a full treatment) would address my previous question regarding how understanding the 1st order question has informed the 2nd order question, while also previewing their forthcoming work.

Several of my previous points remain outstanding. I previously wrote that "many of the justifications for the simplifying assumptions and central tenets of the work are relegated to the appendix - if the journal's restrictions permit, the manuscript would benefit by further substantiating some of these assumptions in the main text.". This appears to be unresolved but I would encourage the authors and editor to follow this recommendation.

Finally, I previously wrote "There are also a few ways in which the rigour of the analysis could be improved, such as in Figure 2(d)-(f) on p5, it would strengthen the argument that the Bayes and DNN histograms are similar if a suitable statistical test showed no significant difference." however the authors have not addressed this point at all in their rebuttal letter or manuscript as far as I can see. Please do so.

References

Evans BD, Malhotra G, Bowers JS. (2022) Biological convolutions improve DNN robustness to noise and generalisation. Neural Networks. 148:96-110. doi: 10.1016/j.neunet.2021.12.005.

Reviewer #4

(Remarks to the Author)
COMMENTS ON THE PREVIOUS ROUND OF REVIEW

I have been asked by the Editor to provide an assessment for the manuscript “Do deep neural networks have an inbuilt Occam’s razor?”, after a previous round of review where the two referees expressed opposite views on the work by Mingard et. al.

In particular, Referee 2 suggests that the present manuscript is “very far away from the bar of Nature Communications”, while Referee 3 recommends it for publication.

As a first, and most important point, I would like to stress that I do not like the attitude of Referee 2. I find their whole report disrespectful towards the authors and lacking of scientific content. Even in the unfortunate case of a rejection, which is a likely outcome in a high-impact journal such as Nature Communication, I think that Referees should not limit themselves to make fun of the work of colleagues, but they should provide constructive criticism to help the authors to improve their manuscript.

SUMMARY OF THE WORK

The manuscript by Mingard and colleagues deals with the important and challenging problem of understanding why deep neural networks generalize well and they do not overfit, as one would expect based on naive parameters counting and on well-established rigorous statistical learning theory results.

The authors investigate this problem using a Bayesian learning perspective, and considering the case of Boolean function classification.

This choice allows them to employ a proxy of the Kolmogorov complexity (whose computation is known to be a NP-hard problem) to test whether an inductive bias towards simple functions, is ultimately the reason for good generalization performance, when combined with data structure.

A crucial ingredient of the authors’ analysis is the observation that (in the case of tanh activation) it is possible to systematically vary the inductive bias over functions tuning a single parameter, i.e. the (common) variance of the Gaussian prior over the weights at each layer.

The authors use this fact as an effective way to probe the complexity of the functions implemented by the network. Their empirical analysis on boolean classification tasks reveals a striking correlation between generalization performance and the complexity of the function learned by the DNN. Finally, they preliminarily employ the same methodology to investigate real-world tasks (non-boolean data), using a different heuristic measure of complexity.

ASSESSMENT

I have mixed feelings about the article by Mingard and colleagues. On one hand, I think their analysis on boolean classification tasks is scientifically sound and reveals a genuine correlation between simplicity and good generalization performance. Moreover, it is in line with the spirit of a physics-driven investigation, as the authors also argue in their reply to Referee 2. On the other hand, I am not entirely sure that what the authors call the “first order generalization problem” (see

end of pag. 1 and first paragraph of pag. 2) can be still considered as relevant as the authors claim, especially in view of the recent analytical results on the infinite-width limit of DNNs (please see comment 1 in the MAJOR COMMENTS paragraph below).

Overall, I will be positively inclined to recommend the manuscript for publication if the authors are able to convincingly address my comments below (in particular major comment 1).

MAJOR COMMENTS

1. In a series of works, which include [1-3], it has been shown that DNNs in the infinite-width limit (with a precise scaling of the weights with width size) are equivalent to kernel learning (respectively the NTK for gradient flow and the NNGP for Bayesian learning). DNNs in this regime are obviously overparametrized, and I would say that the “first order generalization problem” mentioned by the authors is relatively well understood in this case. In particular, all the results found for kernel learning can be immediately translated to large-width networks. For instance, following the seminal paper by Cortes and Vapnik on support vector machines [4], the solutions that generalize better are those with max margin (or equivalently the least-norm weights solutions in the feature space representation of the corresponding constraint satisfaction problem). A statistical physics approach to (polynomial) kernel learning (based on Gardner volumes and replica theory) can be found in [5]. A more recent work on generalization in kernel regression [6] suggests that spectral bias and task-model alignment explain the generalization performance in wide DNNs. It is fair to stress that the authors cite many of the aforementioned papers (mostly in the appendices), but it would be extremely important to have an extended discussion on this related literature in the introduction. How does the present manuscript address issues that are not considered in the previous literature on this topic? What are the key results in this work that are missing from the previous literature and justify the publication in a high-impact journal such as Nature Communications?
2. Numerical experiments shown in the main text are performed only for \sigma_w \geq 1. This value seems somewhat arbitrary. I would recommend the authors to extend their numerical work to smaller values of \sigma_w. In particular, I would be interested in understanding whether the nice correlation shown in Fig. 1 panel (d-f) extend to lower values of \sigma_w.
3. The authors are aware that \sigma_w drives a transition towards a chaotic regime in random DNNs (see their Refs. [22,23]), but they fail to discuss any of the possible implications of this transition in their boolean function classification model. Note that this point has already been addressed for wide DNNs in Ref. [2], where the authors show that the optimal NNGP hyperparameters agree with those predicted by deep signal propagation. Where is the transition to chaos located in their experiments? Does anything special occur at this critical point?

MINOR COMMENTS

1. The authors make a remarkable attempt to summarize the literature on the generalization problem in DNNs in the supplemental material. If possible, I would move most of these references to the main text.
2. Recently, there has been much progress in the Bayesian learning approach to DNNs. References [7,8] propose quantitative theories to move beyond the infinite-width limit, in order to investigate feature learning effects that are not captured by NTK or NNGP kernels. The authors could consider mentioning these papers.
3. For ReLU activation, the simplicity bias barely changes in \sigma_w. Could the authors probe the behaviour of the generalization error as a function of \sigma_w? This might be somewhat related to one of the findings of Ref. [8], where the interplay between the generalization performance and the magnitude of the last layer Gaussian prior is investigated. Please see discussion after their Eq. (8), Fig. 1 panels (b,c) and Fig. 2 panels (c,f).

REFERENCES

[1] Jacot, A., Gabriel, F. & Hongler, C. Neural tangent kernel: Convergence and generalization in neural networks. In Advances in neural information processing systems, 8571–8580 (2018).
[2] Lee, J. et al. Deep neural networks as gaussian processes. arXiv preprint arXiv:1711.00165 (2017).
[3] Matthews, A. G. d. G., Rowland, M., Hron, J., Turner, R. E. & Ghahramani, Z. Gaussian process behaviour in wide deep neural networks. arXiv preprint arXiv:1804.11271 (2018).
[4] C. Cortes & V. Vapnik, Support-vector networks, Machine Learning 20, 273 (1995).
[5] R. Dietrich, M. Opper, & H. Sompolinsky, Statistical mechanics of support vector networks, Physics. Rev. Lett. 82, 2975 (1999).
[6] A. Canatar, B. Bordelon, & C. Pehlevan, Spectral bias and task-model alignment explain generalization in kernel regression and infinitely wide neural networks, Nature Communications 12, 1 (2021).
[7] I. Seroussi, G. Naveh, & Z. Ringel, Separation of scales and a thermodynamic description of feature learning in some CNNs. Nature Communications 14, 908 (2023).
[8] R. Pacelli, S. Ariosto, M. Pastore, F. Ginelli, M. Gherardi & P. Rotondo, A Statistical mechanics framework for deep neural networks beyond the infinite-width limit. Nature Machine Intelligence 5, 1497–1507 (2023).

Version 3:

Reviewer comments:

Reviewer #4

(Remarks to the Author)
Mingard and colleagues provided an extensive and detailed reply to my report, and in particular to my major comment 1,

which I consider crucial to take a decision, as I had already pointed out in my first report.

My overall impression is that the authors did a good job in clarifying my concerns in the reply and in the manuscript, where they have significantly updated both the introduction and the conclusions.
I appreciate this effort, since in the current version it is definitely clear what problem the authors address and are trying to solve.

The introductory paragraph, in particular, now offers a clear perspective of the limitations of previous work on the GP limit of DNNs, making clear the central goal of the manuscript by Mingard et al.

In conclusion, I think that the manuscript in its current form may be of interest for a broad community of statistical physicists, computer scientists and statisticians working in machine learning theory, and I feel it is worth to recommend it for publication in Nature Communications.

Open Access This Peer Review File is licensed under a Creative Commons Attribution 4.0 International License, which permits use, sharing, adaptation, distribution and reproduction in any medium or format, as long as you give appropriate credit to the original author(s) and the source, provide a link to the Creative Commons license, and indicate if changes were made.
In cases where reviewers are anonymous, credit should be given to 'Anonymous Referee' and the source.
The images or other third party material in this Peer Review File are included in the article’s Creative Commons license, unless indicated otherwise in a credit line to the material. If material is not included in the article’s Creative Commons license and your intended use is not permitted by statutory regulation or exceeds the permitted use, you will need to obtain permission directly from the copyright holder.
To view a copy of this license, visit https://creativecommons.org/licenses/by/4.0/

Detailed comments on referee #2 (our comments in red)

This paper tries to argue that the deep neural networks have an inbuilt Occam's razor, from the perspective of Bayesian picture. Specifically, the authors considers the Boolean function setting, where the target function f maps Boolean inputs {0,1}^n to {0,1}, and consider the case with Bayesian priors P(f). They want to understand that if the neural networks are defined on 7 Boolean variables, are 10-layer feedforward network with hidden width 40, and the parameters are randomly sampled, what will happen. Then in Figure 1 and Figure 2, they run experiments to demonstrate some relationships between priors and complexities of the function, as well as how training data affects the posteriors.

Honestly speaking, I think this paper is very far away from the bar of Naturecommunication. First, I am pretty sure that the functions that neural networks learn are not Boolean functions.
This comment is hard to understand. The fact that neural networks can fit and learn Boolean functions is well-known. See 13.3 of "Boolean Models and Methods in Mathematics, Computer Science, and Engineering" CUP (2013) for extensive examples of neural networks fitting Boolean functions. We also show explicitly here that neural networks learn Boolean functions. Of course, if one studies data that is not Boolean, then a DNN will learn other kinds of functions. But Boolean functions are considered to be foundational for a good reason. Boolean data is extremely general, and in this case we are using it as a very general model of classification problems.
Moreover, Boolean functions should take real values for outputs, i.e., [0,1], not {0,1} as assumed in the paper. So the settings the authors considered are extremely restricted.
This statement is extremely odd. It is standard practice to consider Boolean function outputs as discrete, and we use those standard definitions in this paper.
Secondly, the 7 Boolean-variables, 10-layer, 40 hidden width setting is extremely unrealistic, and nobody uses such structure empirically.
We show that nothing fundamental changes if you make the network bigger, and this is very easy to do in practice. In fact we treat the Gaussian process (GP) limit which is equivalent to the infinite width limit, and get very similar results for posteriors. The model-based philosophy that underlies extracting insight for magnetism from a simplified Ising model is the same logic that underlies our choice of a smaller neural network than that typically used by engineering practitioners. Our choice to pare down a DNN is a strength not a weakness. Note that this DNN is provably fully expressive on this dataset, and so can produce all 2^{128}= 3.4 X 10^{38} functions. That remains an enormous number of functions, so this DNN is not small by the standards of the problem we are trying to address. Most importantly, it can easily be made to overfit, which is the conundrum that we are trying to address in this paper.
I think even 8 years ago, such assumptions will be considered extremely strong for submissions to ICML/NeurIPS.
It is true that our style differs from the conventions of big conferences such as ICML/ NeurIPS. But that is, in our opinion, a strength of the paper.
Moreover, this is a purely experimental paper, without any solid evidence of showing why the claims of the authors are true.
This is again very odd. First of all, experiments do provide evidence. Secondly, we do have theory, and show lots of evidence that our theoretical claims are true. This is simply an assertion not backed up by any explanation.
In fact, it is a bit difficult for me to find the exact claims of the authors. Based on the introduction, I think the authors want to demonstrate that the neural network has an inherent selection over simple models during the training process. This is a very important problem, but I do not think this paper provides any convincing answers to it.
We of course disagree with this conclusion, but there is no argument here to engage with, only an assertion.

In the last two pages, the authors also provide some preliminary results on MNIST/CIFAR-10, for experimental results with FCNs. I do not think these results are solid enough to support their claims.

Again, we disagree with this conclusion, but there is no actual argument to engage with here, only an assertion.

To make it clear, I think the main problem of this paper, is the authors picked the wrong title. The correct title should be, “empirically, do 10-layer 40 hidden width feedforward networks on 7 Boolean variables have evidence showing inbuilt Occam’s razor?” I think this is what the paper is really about, which is not significant enough.
Again, this is very odd. We do infinite width limits, for example, and larger networks for MNIST/CIFAR-10 which are, not surprisingly, much harder to fully analyze than the model Boolean problem. This is really a “we don’t do thinks like this in this town” dismissal, it is not an argument.

Detailed response to referee #2 (our comments in red)

This paper tries to argue that the deep neural networks have an inbuilt Occam's razor, from the perspective of Bayesian picture. Specifically, the authors considers the Boolean function setting, where the target function f maps Boolean inputs {0,1}^n to {0,1}, and consider the case with Bayesian priors P(f). They want to understand that if the neural networks are defined on 7 Boolean variables, are 10-layer feedforward network with hidden width 40, and the parameters are randomly sampled, what will happen. Then in Figure 1 and Figure 2, they run experiments to demonstrate some relationships between priors and complexities of the function, as well as how training data affects the posteriors.

Honestly speaking, I think this paper is very far away from the bar of Naturecommunication. First, I am pretty sure that the functions that neural networks learn are not Boolean functions.
The comment starting with "First" is peculiar. The fact that neural networks can fit and learn Boolean functions is well-known, and much studied. See e.g. 13.3 of "Boolean Models and Methods in Mathematics, Computer Science, and Engineering" CUP (2013) for a pedagogical overview. But there are many more articles and book chapters etc… that cover this large topic. We also show explicitly here that neural networks learn Boolean functions. Of course, if one studies data that is not at all Boolean, then a DNN will learn different kinds of functions. But Boolean functions are considered to be foundational in many fields for good reasons. Boolean data is extremely general, and in this case, we are using it as a generic model of classification problems. **The great advantage of Boolean data is that one can finely control the complexity of the target function, something that is much harder to do with many other model datasets, e.g. standard image classification.**

Moreover, Boolean functions should take real values for outputs, i.e., [0,1], not {0,1} as assumed in the paper. So the settings the authors considered are extremely restricted.
This statement is also peculiar. It is standard practice to consider Boolean function outputs as discrete, and we use those standard definitions in this paper.
Secondly, the 7 Boolean-variables, 10-layer, 40 hidden width setting is extremely unrealistic, and nobody uses such structure empirically.

We are somewhat mystified by this referee report. We surmise that at the root of this referee's overall response may arise from **fundamental philosophical difference of approach**. In engineering practice, one does indeed use much larger models, and this is necessary to obtain the kind of state-of-the-art performance that has so energized the community. But here we are looking at a different foundational problem. In our paper we write on page 2:

``Before proceeding, it is important to distinguish the question above from a different and equally interesting question: Given a DNN that generalizes reasonably well (e.g. it solves the overparameterization/large capacity problem), can we understand how to improve its performance further? This 2nd-order question is what practitioners of deep learning typically care about. Differences in architecture, hyperparameter tuning, data augmentation etc. can indeed lead to important improvements in DNN performance. Exactly why these tweaks and tricks generate better inductive bias is typically not well understood either, and is an important subject of investigation. Be- cause the two questions are often conflated, leading to confusion, we want to emphasise up front that this paper will focus on the 1st-order conundrum shared by all over- parameterized DNNs. Understanding this basic problem should help frame important 2nd-order questions about how to improve DNN performance further"

For the 2nd order problem that engineers care about large DNNs are needed and improving generalisation may hinge on the addition of certain complexities. However, for the 1st order problem, shared by all DNNs that we are treating here, that added complexity is, we argue, not necessary. The philosophy of our approach, which is very common in many scientific fields, is to study **a model that**

is stripped of details that are not necessary to explain the phenomenon one is trying to understand. A classic example would be the Ising model of magnetism, which is much simpler than real magnetic materials, but captures their essense beautifully. In his 1995 paper "Reflections After Refereeing Papers for NIPS" The Mathematics of Generalization, 11–15, Leo Breiman also directly makes the conceptual link to this model, calling for

- simplified analogies (like the Ising Model)

in order to make progress on questions such as 1st order problem above, which he calls one of the key unanswered questions in the field. In a recent article, Understanding deep learning is also a job for physicists. Nature Physics **16**, 602 (2020) Lenka Zdeborová also mentions the need for Ising model like approaches and says:

> "In particular, the theoretical part of physics research is largely based on models. Models are a way of capturing the essence of a problem and stripping off the details that are not necessary to explain the experimental observations. An example would be the widely used Ising model of magnetism"

In our paper we are taking exactly this kind of approach. Our Boolean systems are more akin to the Ising model, than they are to the latest state of the art engineering model. The fact that one of the key fundamental questions that Breiman raised, namely "Why don't heavily parameterized neural networks overfit the data?" is unanswered 28 years later should tell us that new approaches are desperately needed in this field.

We write on page 2

Just as the Ising model does for magnetism, this simple but versatile model allows us to capture the essence of the overparameterization problem, while remaining highly tractable.

In other words, we explicitly state our intent early on, the ``philosophy'' we are using here is not somehow a hidden assumption. For that reason, it is all the more surprising that the referee apparently hasn't picked up on this.

Secondly, and more trivially, we also tried larger DNNs and explicitly show that nothing fundamental changes if you make the network bigger. In fact, we treat the Gaussian process (GP) limit which is equivalent to the infinite width limit, and get very similar results for posteriors. In other words, **Our choice to pare down a DNN is a strength not a weakness.**

Thirdly, note that this DNN is provably fully expressive on this dataset, and so big enough to produce all 2^{128}= 3.4 X 10^{38} functions. For example, if ½ of the 128 inputs are in the training set, there remain 2^{64}=10^{19}distinct functions that give zero training error. These numbers are huge! Of course they are even bigger in larger systems, but for questions such as "Which of the 2^{64} " zero training error functions will a DNN converge to?", these numbers are large enough to capture the essence of the 1st order generalisation problem. Most importantly, we show that such DNNs can also easily be made to overfit. So why don't they do so in practice? So Breiman's conundrum is fully present in our model problem.
I think even 8 years ago, such assumptions will be considered extremely strong for submissions to ICML/NeurIPS.
It is true that our style differs from the conventions of big conferences such as ICML/ NeurIPS. But that is, in our opinion, a strength of the paper, see also the discussion above.
Moreover, this is a purely experimental paper, without any solid evidence of showing why the claims of the authors are true.

First of all, if it was purely an experimental paper, then the question should be: do the experiments show evidence for the hypothesised claims. We claim that our experiments do just that. What is doubly peculiar about this sentence is that we do provide lots of theory, e.g. bounds, scaling laws etc… It is hard to respond to claims that do not reflect the paper.
In fact, it is a bit difficult for me to find the exact claims of the authors. Based on the introduction, I think the authors want to demonstrate that the neural network has an inherent selection over simple models during the training process. This is a very important problem, but I do not think this paper provides any convincing answers to it.
We simply disagree –we would love to engage in detail with this complaint, but there is unfortunately nothing of substance here to engage with.

In the last two pages, the authors also provide some preliminary results on MNIST/CIFAR-10, for experimental results with FCNs. I do not think these results are solid enough to support their claims.

As argued above, one of the great strengths of Boolean data is that one can control and measure the complexity of the data. That is harder for image datasets. Nevertheless, we show that the basic behaviours we observe in the better controlled model problems also obtain in these larger scale systems. Such extrapolations are the essence of the model based philosophy we are using here. It is hard to know how one could do much better than this for these more complex systems.

To make it clear, I think the main problem of this paper, is the authors picked the wrong title. The correct title should be, "empirically, do 10-layer 40 hidden width feedforward networks on 7 Boolean variables have evidence showing inbuilt Occam's razor?" I think this is what the paper is really about, which is not significant enough.
The referee has missed that we do the infinite width limit and show that these give the same results as our stripped-down model problem, which remains provably fully expressive on this problem. We also did larger DNNs, but the differences were minor, as expected. The same effects obtain on larger Boolean datasets, but of course with less nice statistics. We also study larger networks for MNIST/CIFAR-10 which are, not surprisingly, much harder to fully analyze than the model Boolean problem. It is true that our approach – using a stripped down model problem, and then extrapolating up to larger scale systems – is not the kind of work you see much of in traditional conferences such as ICML/NeurIPS, but, given that the very basic and fundamental 1st order problem of generalisation is still not solved, new approaches should be welcomed, not dismissed.

Our response has been lengthy, mainly because we are guessing at what the referee is thinking. Given that they missed the fact that we used the infinite width limit GPs to calculate posteriors, we have now expanded our discussion of that work in the hope that this will make it harder to miss (top of page 6, column 2).

Reviewer #3 (Remarks to the Author):

This manuscript makes a mathematical argument, couched in a Bayesian framework, for Deep Neural Networks (DNNs) having an inductive bias such that the functions they are predisposed to learn shrink in probability exponentially with increasing complexity, which counteracts the exponential growth in the space of possible functions as their complexity grows. This argument is instantiated with simulations demonstrating the effect, measured in terms of the probability of learning a particular function and the generalisation error, on some toy model architectures applied to simple boolean and standard benchmark datasets (i.e. CIFAR-10 and MNIST), as some of their key parameters, such as depth, are systematically varied.

This is a noteworthy and insightful result shedding light on a complex and powerful property of DNNs, relevant to the perplexing findings identified in such work as Nakkiran et al., 2019 (https://arxiv.org/abs/1912.02292); a publication which this manuscript would benefit from citing.

We thank the referee for the positive comments, and for suggesting that we cite e Nakkiran et al paper on double descent, which we now do on page 1 of the appendix, where we write

"Furthermore, more subtle double descent effects with respect to the quantity of data, training epochs and model size have been observed in e.g. \cite{nakkiran2021deep}".

The approach appears to be methodologically sound, however many of the justifications for the simplifying assumptions and central tenets of the work are relegated to the appendix - if the journal's restrictions permit, the manuscript would benefit by further substantiating some of these assumptions in the main text.

We put the highlights in the main text, and relegated the technical details to the Appendix. Of course, if the editors allow it, we would be delighted to put key sections from the Appendix into the main text

Regarding the separate question of how to improve the generalisation of DNNs, the authors state: "Understanding this basic problem should help frame important 2nd-order questions about how to improve DNN performance further." While they take care to disentangle these questions in the introduction, having arrived at an understanding of the basic problem, the authors do not discuss this aspect, which would be useful for the large proportion of readers interested in the "second-order question" - how has their finding helped to frame such questions and what insights into how to further improve DNN performance has it or could it provide?

This is an excellent question – we believe that most of the 2nd order improvements are linked to "feature learning", something that, for example, infinite width DNNs that reduce to Gaussian Processes do not do. We are currently writing a long paper on feature-learning where we explore how the bias towards simplicity helps us understand 2nd order improvements, but the story is complex enough that we can't easily summarize or put it into the present paper. What also makes theses 2nd order improvements harder to analyze is that they are always there in stripped down model systems. In some cases (not all) the added complexity of a larger model is critical.

There are also a few ways in which the rigour of the analysis could be improved, such as in Figure 2(d)-(f) on p5, it would strengthen the argument that the Bayes and DNN histograms are similar if a suitable statistical test showed no significant difference.

There are also a few small mistakes and typos which should be corrected, as follows.

* "its argument is true if the parameters of N (Θ) are such that represents f It was shown in [10] that", p2: this is ungrammatical and missing a full stop.
* Please make the ordering of items in the legends consistent e.g. 1(a) and (b), p3.
* "PAC bound" p4: Define acronyms on first use.
* "(with confidence $0 \geq (1 - \delta) \leq 1$)" p4: the first inequality appears to be reversed.

* "(Eq. (3) captures the dominant trends...": Missing bracket in Fig 2, p5.
* "Beyond the Boolean model: MINST & CIFAR-10", p6: Typo in MNIST.
* "2nd order question of fine-tuned generalization": There is a formatting anomaly half way down p2 of the Supplementary materials.

Many thanks for catching these, they have all been fixed now.

Overall, I find this to be an important analysis of DNNs and subject to the revisions above, I recommend it for publication.

We thank the referee for their final recommendation.

RESPONSE TO REFEREES

We apologise to the referees for the delay in our response to their comments. This was mainly caused by illness in our group.

REVIEWER COMMENTS

Reviewer #2 (Remarks to the Author)

I have read the authors' rebuttal. However, I did not change my mind.

1. Sorry, I still do not think neural network can be approximated with Boolean functions. I have read many theory papers on deep learning, and I have never seen such assumptions previously. I think this is a very strong assumption that I cannot accept.

We think the puzzling report by the referee may be due to some fundamental misunderstandings. We are not approximating a neural network with Boolean functions, but learning Boolean functions with a neural network.

2. Sorry, Boolean functions take real values. Please check https://arxiv.org/abs/2105.10386 by Ryan O'Donnell. It is standard to consider Boolean function outputs as real value, not discrete value.

Page 19 of this recommended piece begins by stating the following

1.1. On analysis of Boolean functions

This is a book about Boolean functions,

$$f : \{0,1\}^n \to \{0,1\}.$$

Here f maps each length-n binary vector, or *string*, into a single binary value, or *bit*. Boolean functions arise in many areas of computer science and mathematics. Here are some examples:

Which contradicts what the referee is claiming.

3. Sorry, I think the 7 Boolean-variables, 10-layer, 40 hidden width setting is extremely unrealistic, and nobody uses such structure empirically. I am afraid that I cannot agree with the authors that this case captures enough details of the topic.
Again, it captures SOME details on the topic.

I also read the rest of the rebuttal, and did not change my mind.

Therefore, I will maintain my score unchanged. I think this paper is very far away from being published at Nature communications.

As we wrote in our previous reply, there is always a tradeoff between studying simpler models that are more tractable v.s. large-scale engineering models where many confounding factors complicate analysis. We chose the model problem approach because the particular conundrum we are addressing is fully present there in a clear way. Nevertheless, we have now included a much larger DNN with 128 Boolean variables and 2^(2^128) functions (Fig S16), which shows similar behaviour to the smaller more tractable DNNs.

Reviewer #3 (Remarks to the Author)

As per my previous review, I consider that this article addresses an interesting and important point in a rigorous and controlled way. Unlike the other reviewer, I like the authors' approach of paring the problem down to a toy problem (at least, relative to the cutting edge of the datasets DNNs are typically trained on) but one that is tractable, finely controllable and in their own words "foundational". Indeed, it is hard to argue that boolean logic is anything but foundational in computer science.

However, to help bridge the gap between the experimental petri disk of boolean functions and the more complex problems of engineering applications, (in order to counter the objections such as those faced in this review process from Rev. 2.) it would help to give more prominence to the studies (in the main text) where the authors scaled the problem up and found similar results for the basic behaviours.

To further assuage such objections, the introduction could be slightly expanded with citations to more applied work on inductive biases in DNN. For example, in a biologically-inspired convolutional neural network, less was also shown to be more (i.e. through simplifications/constraints conferring benefits) in work by Evans et al. (2022) whereby fixing the form of the first layer convolutional kernels led to greater robustness to noise and generalisation on out-of-distribution images. Inductive biases of this sort found through evolution may similarly have been implicitly found by engineers tinkering with neural networks (prior to this analytical perspective), contributing to the enormous popularity and success they have enjoyed.

A brief summary of the central hypothesis of the authors' forthcoming paper on feature learning could also be included in this section. I appreciate that the full story can not be included in the current paper but a sentence or two to at least articulate the central question and sketch out their idea (citing the manuscript in preparation for a full treatment) would address my previous question regarding how understanding the 1st order question has informed the 2nd order question, while also previewing their forthcoming work.

Several of my previous points remain outstanding. I previously wrote that "many of the justifications for the simplifying assumptions and central tenets of the work are relegated to the appendix - if the journal's restrictions permit, the manuscript would benefit by further substantiating some of these assumptions in the main text.". This appears to be unresolved but I would encourage the authors and editor to follow this recommendation.

We thank the referee for the helpful comments and suggestions in the paragraphs above. We have taken these on board in the following way:

On page 2 we added about a ½ page (in red in the document) of broader discussion regarding questions 1 and 2 on generalisation. We have brought in parts from the Appendix into the main text to clarify the points raised above. We are constrained by length, and hope that a reader who wants more background can still turn to the Appendices.

We have also substantially rewritten the conclusions sections (also in red in the new text) to address a number of the points raised by the referee. For example, we now cite the very interesting Evans et al (2022) paper, which we had not seen before, in the context of an expanded discussion of 1st and 2nd order generalisation. Nevertheless, the main point of our paper is the simpler 1st order question, and we are space-limited in how far we can discuss the much more complex question of how improved inductive biases may help explain why a particular DNN, trained in a particular way does best on a particular dataset (e.g. why are transformers better than LSTMs for large language modelling, or why is a ResNET usually (but not always) better than a CNN on Imagenet, or why does a larger learning rate typically generalise slightly better for many optimisers etc…). These are hard problems to say anything general about without opening up many other cans of worms.

Our paper on feature-learning does the following: We measure changes in features by calculating the eigenfunctions of the final layer, and show that under SGD and on classification tasks with C labels, many DNNs collapse those down to a minimal set of C eigenfunctions, instead of using the full set of L functions, where L is the width of the final layer. We call this the minimum feature regime (MFR) and its behaviour resembles neural collapse (NC) upon overtraining. In other cases, there is still a reduction in the effective number of eigenfunctions used, but the number is larger than C, and smaller than L. We call this the extended feature learning regime (EFR). The infinite-width limit where there is no feature-learning corresponds to using all L eigenfunctions as they are set at initialisation, and simply learning the coefficients. Typically this does not generalise quite as well as the MFR or EFR regimes, but all three regimes generalise reasonably well and so solve the 1st order problem of learning in the overparameterized regime. Here, the feature-learning acts as a 2nd order effect. We find it all super interesting, but it is beyond the scope of this paper.

Finally, to scale our problem up, we performed calculations on a Boolean problem with n=128, which is a much bigger problem - the total set of possible inputs is 2^(128) \approx 3 \times 10^(38), and the number of possible functions is 2^2^(128). We observe that a normal DNN can generalise on simple functions, but not on complex ones, while DNNs with slightly larger \sigma_w don't generalise at all. None of the DNNs can generalise on complex functions. This can all be seen in supplementary Figure S16.

Finally, I previously wrote "There are also a few ways in which the rigour of the analysis could be improved, such as in Figure 2(d)-(f) on p5, it would strengthen the argument that the Bayes and DNN histograms are similar if a suitable statistical test showed no significant difference." however the authors have not addressed this point at all in their rebuttal letter or manuscript as far as I can see. Please do so.

We have now added a Fig S.22 in the appendices which provides a series of statistical tests and comparisons of averages and standard deviations.

References

Evans BD, Malhotra G, Bowers JS. (2022) Biological convolutions improve DNN robustness to noise and generalisation. Neural Networks. 148:96-110. doi: 10.1016/j.neunet.2021.12.005.

Reviewer #4 (Remarks to the Author)

COMMENTS ON THE PREVIOUS ROUND OF REVIEW

I have been asked by the Editor to provide an assessment for the manuscript “Do deep neural networks have an inbuilt Occam’s razor?”, after a previous round of review where the two referees expressed opposite views on the work by Mingard et. al.

In particular, Referee 2 suggests that the present manuscript is “very far away from the bar of Nature Communications”, while Referee 3 recommends it for publication.

As a first, and most important point, I would like to stress that I do not like the attitude of Referee 2. I find their whole report disrespectful towards the authors and lacking of scientific content. Even in the unfortunate case of a rejection, which is a likely outcome in a high-impact journal such as Nature Communication, I think that Referees should not limit themselves to make fun of the work of colleagues, but they should provide constructive criticism to help the authors to improve their manuscript.

SUMMARY OF THE WORK

The manuscript by Mingard and colleagues deals with the important and challenging problem of understanding why deep neural networks generalize well and they do not overfit, as one would expect based on naive parameters counting and on well-established rigorous statistical learning theory results.

The authors investigate this problem using a Bayesian learning perspective, and considering the case of Boolean function classification.

This choice allows them to employ a proxy of the Kolmogorov complexity (whose computation is known to be a NP-hard problem) to test whether an inductive bias towards simple functions, is ultimately the reason for good generalization performance, when combined with data structure.

A crucial ingredient of the authors’ analysis is the observation that (in the case of tanh activation) it is possible to systematically vary the inductive bias over functions tuning a single parameter, i.e. the (common) variance of the Gaussian prior over the weights at each layer.

The authors use this fact as an effective way to probe the complexity of the functions implemented by the network. Their empirical analysis on boolean classification tasks reveals a striking correlation between generalization performance and the complexity of the function learned by the DNN. Finally, they preliminarily employ the same methodology to investigate real-world tasks (non-boolean data), using a different heuristic measure of complexity.

ASSESSMENT

I have mixed feelings about the article by Mingard and colleagues. On one hand, I think their analysis on boolean classification tasks is scientifically sound and reveals a genuine correlation between simplicity and good generalization performance. Moreover, it is in line with the spirit of a physics-driven investigation, as the authors also argue in their reply to Referee 2. On the other hand, I am not entirely sure that what the authors call the “first order generalization problem” (see end of pag. 1 and first paragraph of pag. 2) can be still considered as relevant as the authors claim, especially in view of the recent analytical results on the infinite-width limit of DNNs (please see comment 1 in the MAJOR COMMENTS paragraph below).

Overall, I will be positively inclined to recommend the manuscript for publication if the authors are able to convincingly address my comments below (in particular major comment 1).

MAJOR COMMENTS

1. In a series of works, which include [1-3], it has been shown that DNNs in the infinite-width limit (with a precise scaling of the weights with width size) are equivalent to kernel learning (respectively the NTK for gradient flow and the NNGP for Bayesian learning). DNNs in this regime are obviously overparametrized, and I would say that the “first-order generalization problem” mentioned by the authors is relatively well understood in this case. In particular, all the results found for kernel learning can be immediately translated to large-width networks. For instance, following the seminal paper by Cortes and Vapnik on support vector machines [4], the solutions that generalize better are those with max margin (or equivalently the least-norm weights solutions in the feature space representation of the corresponding constraint satisfaction problem). A statistical physics approach to (polynomial) kernel learning (based on Gardner volumes and replica theory) can be found in [5]. A more recent work on generalization in kernel regression [6] suggests that spectral bias and task-model alignment explain the generalization performance in wide DNNs.

It is fair to stress that the authors cite many of the aforementioned papers (mostly in the appendices), but it would be extremely important to have an extended discussion on this related literature in the introduction. How does the present manuscript address issues that are not considered in the previous literature on this topic? What are the key results in this work that are missing from the previous literature and justify the publication in a high-impact journal such as Nature Communications?

We thank the referee for encouraging us to clarify this point. To that end, we have now added an expanded section to the introduction (in red and mainly on page 2) and also a brief recap of these points in the conclusion (also in red). We have kept the longer discussion of

this literature in the Appendix for those who want a more in-depth discussion (Someone should write a review on this b.t.w.) We know this literature well, since one of us has published in this direction, see our refx[22], El Harzli et al, that uses random matrix theory to calculate generalisation measures for a GP.

The recent literature on task-model alignment (TMA) provides quantitative measures of generalisation in terms of eigenvalues and eigenvectors of a kernel. To obtain a low generalisation error, the target function should be expressible by a small number of eigenfunctions with large eigenvalues. In this way, the TMA quantifies whether or not the model has a good inductive bias for the task. Since these kernels and GPs are inspired by DNNs, and give similar generalisation errors, at least on more basic model data, it is widely thought that these calculations also provide insight into what DNNs are doing. However, while these methods tell us ***how*** to calculate the generalisation error, they don't provide much (yet) in terms of answering ***why*** a task and a model are or are not aligned, e.g. why or why not do you only need a small number of eigenfunctions to describe a particular target function.

To answer broader why questions, one needs to analyse not only what functions a learner converges to, but also the counterfactual space of functions that it could have converged to, but didn't.

Our key result, that the inductive bias of the prior scales as 2^(-K), and so counteracts the 2^K growth of the number of functions with complexity, coupled with our arguments that this prior helps explain the posterior of trained DNNs, provides a key global "why" ingredient for answering Breiman's 1995 question. TMA results provide a different kind of complementary insight at a finer-grained level. We therefore believe that our key results are new and of sufficient interest to warrant publication in Nature Communications.

It would also be extremely interesting to expand the set of techniques used to derive TMA measures and attempt to answer a similar global "why" question in the context of the regression setting for continuous functions that these methods treat. Indeed, ever since we derived our own TMA-like measures for GPs using random matrix theory techniques (see our ref [22]), we have been trying to achieve something of this nature. We have not been successful yet. We can semi-analytically calculate what the generalisation error will be in certain settings in terms of eigenfunctions and eigenvalues, but we don't understand fundamentally why the error is large or small. Part of the difficulty is that the setting of continuous functions makes it harder to do the kind of global examination of the function space that would be needed.

Finally, we agree with the referee that TMA falls under the category of trying to understand question 1. These approaches are typically applied models that are not trained by SGD, and so many of the tricks and hyperparameter tunings that lead to the best possible generalisation by DNNs are not available. Moreover, these models do not show feature learning (see our other response above), which we argue elsewhere is a key ingredient for improving generalisation further.

As an addendum, we note that we have also added fig S17, which shows how different learners lead to different PAC-Bayes generalisation bounds for Fig 1c. These bounds complement our results on the posteriors.

2. This value seems somewhat arbitrary. I would recommend the authors to extend their numerical work to smaller values of \sigma_w. In particular, I would be interested in understanding whether the nice correlation shown in Fig. 1 panel (d-f) extend to lower values of \sigma_w.

We thank the referee for this suggestion. In Supplementary figures S6 and S10 we now show priors over functions and complexity, as well as posterior scatter plots for the ordered regime with small \sigma_w. We treat a number of different values, down to the limit of very small \sigma_w where the DNN reduces to a linear network.

3. The authors are aware that \sigma_w drives a transition towards a chaotic regime in random DNNs (see their Refs. [22,23]), but they fail to discuss any of the possible implications of this transition in their boolean function classification model. Note that this point has already been addressed for wide DNNs in Ref. [2], where the authors show that the optimal NNGP hyperparameters agree with those predicted by deep signal propagation. Where is the transition to chaos located in their experiments? Does anything special occur at this critical point?

We discuss the transition to chaos in Appendix C. It occurs in the limit of infinite depth, and then one must use $\sigma_w=1$ to be on the “edge of chaos”, for tanh activations. For smaller finite depths there is a broader cross-over regime which is what we show in our Fig 1 and Fig S6 for example, and so $\sigma_w=1$ is not needed to be able to train. In Fig S6, we observe that P(K) is still approximately flat for $\sigma_w < 1$ and for that depth (10 layers) it only appreciably starts to deviate when $\sigma_w=1.5$. For larger depths, this deviation would occur for σ closer to 1.

MINOR COMMENTS

1. The authors make a remarkable attempt to summarize the literature on the generalization problem in DNNs in the supplemental material. If possible, I would move most of these references to the main text.

We have moved a good number of the references to the main text, but there are length limitations that mean we have kept some material in the Appendices.

2. Recently, there has been much progress in the Bayesian learning approach to DNNs. References [7,8] propose quantitative theories to move beyond the infinite-width limit, in order to investigate feature learning effects that are not captured by NTK or NNGP kernels. The authors could consider mentioning these papers.

We thank the referee for pointing out these very interesting papers and we now discuss them in our rewritten conclusion section.

3. For ReLU activation, the simplicity bias barely changes in \sigma_w. Could the authors probe the behaviour of the generalization error as a function of \sigma_w? This might be somewhat related to one of the findings of Ref. [8], where the interplay between the generalization performance and the magnitude of the last layer Gaussian prior is investigated. Please see discussion after their Eq. (8), Fig. 1 panels (b,c) and Fig. 2 panels (c,f).

We now include in Figure S10 and S11 some results for RELU activations with a range of \sigma_w values. There are some modest changes in generalisation as a function of \sigma_w, but we haven't been able to quite make the connection to Eq (8) and Figs 1 and 2 of ref[8]. We will continue to investigate this matter though. The setting in [8] uses a mean square error-like likelihood. Figures S10 and S11 show that, when using mse loss, generalisation is worse for larger sigma_w, for both ReLU and tanh activations. When using cross-entropy loss, ReLU still performs well even as sigma_w increases. This is because the exact scale of the weight initialisation in the last layer does not affect networks trained with cross-entropy loss as strongly as those with mse loss (as the target scale is less important).

REFERENCES

[1] Jacot, A., Gabriel, F. & Hongler, C. Neural tangent kernel: Convergence and generalization in neural networks. In Advances in neural information processing systems, 8571–8580 (2018).
[2] Lee, J. et al. Deep neural networks as gaussian processes. arXiv preprint arXiv:1711.00165 (2017).
[3] Matthews, A. G. d. G., Rowland, M., Hron, J., Turner, R. E. & Ghahramani, Z. Gaussian process behaviour in wide deep neural networks. arXiv preprint arXiv:1804.11271 (2018).
[4] C. Cortes & V. Vapnik, Support-vector networks, Machine Learning 20, 273 (1995).
[5] R. Dietrich, M. Opper, & H. Sompolinsky, Statistical mechanics of support vector networks, Physics. Rev. Lett. 82, 2975 (1999).
[6] A. Canatar, B. Bordelon, & C. Pehlevan, Spectral bias and task-model alignment explain generalization in kernel regression and infinitely wide neural networks, Nature Communications 12, 1 (2021).
[7] I. Seroussi, G. Naveh, & Z. Ringel, Separation of scales and a thermodynamic description of feature learning in some CNNs. Nature Communications 14, 908 (2023).
[8] R. Pacelli, S. Ariosto, M. Pastore, F. Ginelli, M. Gherardi & P. Rotondo, A Statistical mechanics framework for deep neural networks beyond the infinite-width limit. Nature Machine Intelligence 5, 1497–1507 (2023).
